# Patient views on use of emergency and alternative care services for adult epilepsy: A qualitative study

**DOI:** 10.1016/j.seizure.2020.04.011

**Published:** 2020-08

**Authors:** Alison McKinlay, Myfanwy Morgan, Adam Noble, Leone Ridsdale

**Affiliations:** a King's College London, Department of Basic and Clinical Neuroscience, London, UK; b King’s College London, Institute of Pharmaceutical Science, London, UK; c University of Liverpool, Department of Health Services Research, UK

**Keywords:** ACP, alternative care pathway, ENS, epilepsy nurse specialist, UTC, urgent treatment centre, Self-management, Self-care, Patient care planning, Epilepsy, Emergency department

## Abstract

**Purpose:**

Emergency Department (ED) visits are costly to the health service and alternative care pathways may address this whilst improving outcomes. We aimed to describe decision-making and preferences of people with epilepsy (PWE) during emergency service use, and views of ED alternatives, including use of an Urgent Treatment Centre and telephone-based support from an epilepsy nurse specialist.

**Methods:**

We conducted a community-based interview study in South East England, informed by a qualitative framework approach. 25 adults with epilepsy and 5 of their carers took part.

**Results:**

Participants’ choice to attend ED generally corresponded with guidelines, including continuing seizures and injury. Nevertheless, over half reported unwanted or unnecessary ED attendance, mainly due to lack of access to individual patient history, a carer, or seizures occurring in a public place. Participants used proactive strategies to communicate their care needs to others, including 24 -h alarm devices and care plans. Some suggested preventative strategies including referral after ED. Participants highlighted the importance of ambulance staff in providing fast and efficient care that gives reassurance.

**Conclusion:**

Improving communication and access to preventative, proactive services may facilitate better outcomes within existing care pathways. PWE felt ED alternatives were helpful in some circumstances, but Urgent Treatment Centres or epilepsy nurse specialists were not viewed as an ED replacement.

## Highlights

• We interviewed highly informed people with epilepsy and their carers
• Participants usually preferred to avoid emergency services after a typical seizure
• They developed strategies to manage themselves without emergency services
• Improved communication between existing services will facilitate appropriate care
• Two alternative pathways were not viewed as replacements for emergency care

## 1 Introduction

Around 20% of people with epilepsy (PWE) visit a hospital emergency department (ED) each year [[1], [2], [3], [4]]. Nine out of 10 PWE arrive at ED by ambulance [5,6]. Such visits are expensive, with around half of them resulting in admission [7]. The annual cost to the National Health Service (NHS) for these visits in England alone is ∼£70–90 M [[7], [8], [9]].

Emergency care for epilepsy can be important, even lifesaving (e.g., a first seizure, status epilepticus, significant injury [10,11]). However, there has been increasing interest in ED attendance by PWE, because their visits are often not for these reasons and appear to be clinically unnecessary [[12], [13], [14]]. The National Audit of Seizure Management in Hospitals (NASH) [15] and others [7] indicate most attendees have diagnosed epilepsy and have experienced an uncomplicated seizure. NASH found ED visits for epilepsy typically lead to few improvements in epilepsy management. In the UK, most PWE are managed by their general practitioner (GP) with a minority referred back to a specialist to monitor a change in care needs (e.g., sezure control). NASH found that most PWE visiting ED were not known to a specialist when they arrived, and going to ED did not change this, despite evidence that they might have been helped by such input. It is therefore not surprising that ∼60% of PWE attending ED reattend in the next 12 months [4].

Although ∼70% of PWE should become seizure-free with optimal treatment, [16] evidence suggests seizure-freedom is achieved by around 50% of PWE in the UK [17]. The NHS – like many other health systems around the world – is committed to improving efficiency and productivity, whilst driving up care quality, reducing health inequalities, and improving outcomes [18,19]. One way to achieve this is by developing innovative models of care delivery, for example, introducing alternative care pathways (ACPs) that ambulance crews might use, where appropriate, to divert adults with epilepsy away from ED, to be cared for elsewhere [20]. This might be at home, with follow-up care provided by an epilepsy nurse specialist (ENS) within 24 h, or a so-called Urgent Treatment Centre (UTCs – see Appendix A Supplementary Data).

**Fig. 1:**
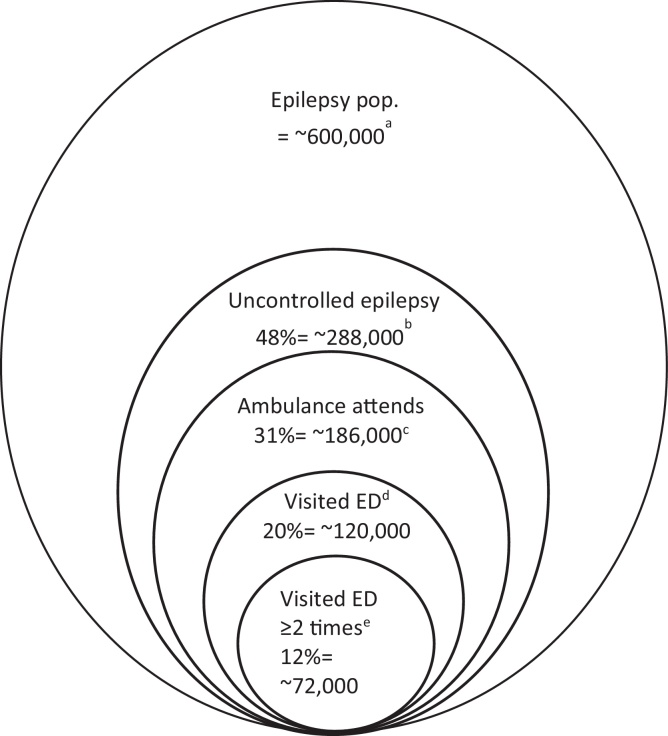
Depiction of ambulance service use and Emergency Department attendance by people with epilepsy in UK. Notes: Not to scale; ^a^ Based on ∼1 % of UK population having epilepsy; [29] ^b^ Moran et al. indicate 48 % of people with epilepsy will have had a seizure in prior 12 months; [17] ^c^ When a call is received by a regional ambulance service that is described as relating to a ‘convulsion’, ‘fit’, ‘seizure’, the call handler will endeavour to ask standardised questions to gauge, such things as severity and potential aetiology. For services using the Advanced Medical Priority Dispatch System (AMPDS), according to Protocol 12, one question is “Is s/he an epileptic?”. Most callers should be able to answer question as most (∼70 %) seizure calls are made by a relative, friend or carer [12]. Audit data from two regional ambulance services (North-West Ambulance Service, personal communication, Head of Research and Development, 29^th^ July 2019; Yorkshire Ambulance Service, personal communication, Head of Service Development Emergency Operation Centre, 30th May 2019) indicates that in 2018 of those attended to who were recorded as having a history of epilepsy according to the AMPDS screening question, ∼70 % were conveyed to ED and 30% were not; ^d^ Hart and Shorvon found ∼20 % of PWE reported attending an ED in the prior 12 months; [30] ^e^ Noble et al. found ∼60 % of PWE reattend within 12 months.

With any change to services, we need the views of patients and those supporting them [21]. The acceptability of an intervention to its intended recipients is a fundamental criterion for it to be well positioned to achieve its intended outcome (see ‘APEASE’ framework) [[22], [23], [24], [25]]. The views of PWE and their family and friends (to whom care decisions are often delegated) have not yet been sufficiently explored in detail [26], yet, there are indicators that their views can vary substantially. Around 50% of PWE in the UK experience a seizure in the past year, but less than half attend ED (Fig. 1) [17]. This includes people who do not not seek emergency care in the first instance, and others declining a visit ED even after an ambulance has attended [27,28]. The aim of the current study was therefore to elicit PWE and carer experiences and preferences for emergency service care following a seizure. We sought to answer the following research questions: i) What are patient and carers’ decision-making processes for seeking or not seeking ED care when a seizure occurs? ii) What are their concerns and expectations regarding alternative care approaches?

## 2 Methods

### 2.1 Study design

A qualitative interview study was undertaken to provide a detailed account of PWEs’ views about emergency care, as part of our ongoing research to develop patient-centred, feasible alternative care options for adult ED users with epilepsy [31]. This study received ethics approval by the King’s College London Psychiatry, Nursing and Midwifery Ethics Committee (LRS-18/19-10353). Methods were guided by the COREQ checklist [32] for reporting qualitative research.

### 2.2 Recruitment

‘Appendix A Supplementary Data’ contains full eligibility criteria. In brief, eligible participants were aged ≥18, had a self-reported diagnosis of epilepsy for ≥1 year, with emergency service contact – be it a visit/s to ED and/ or attendance/s by the ambulance service – in the previous 12 months for epilepsy. No medical records were accessed at any time to confirm diagnosis or ED attendance. Given that decisions regarding care can be delegated when the patient is unconscious or lacks capacity [34], participants with epilepsy were invited to bring a family member or friend to their interview to participate as a “carer” (defined as: someone who provides support with their epilepsy). PWE were reimbursed £20 for their time. Five carers participated during interviews (parent: 3, child: 1, partner: 1).

Purposive recruitment methods were used. The first author (AM) invited by mail 47 PWE from a previous randomised controlled trial [33] who agreed to be contacted about future research. Fifteen PWE responded to the invitation and were screened; eight of whom were eligible to participate. Epilepsy Action, a user-led charity in the UK, also assisted with the recruitment of 17 participants by advertising the study through social media, community events and newsletters. Interested PWE were invited to phone or email the research team to register their interest. Of the 37 people who made contact, 21 were eligible after screening, of whom 4 dropped out due to non-response to interview requests or non-attendance. Recruitment concluded at 25 interviews, as no new themes were identified.

### 2.3 Procedure

**Fig. 2:**
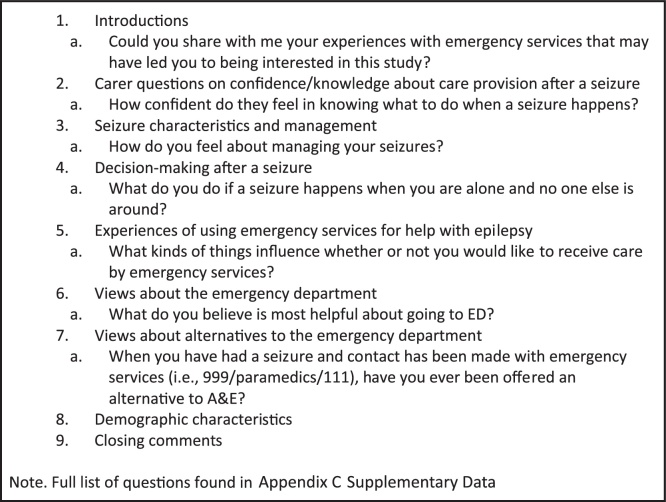
Interview Topic Guide and Examples.

An interview guide was developed by all study authors (two research psychologists, one neurologist, and one medical sociologist), with involvement from Epilepsy Action, who reviewed the language in participant documents and interview topics. Interview guides were reviewed by two PWE and one ambulance staff member, who provided insight based on their experience. The resulting topic guide (briefly described in Fig. 2, full version in Appendix A Supplementary Data) and procedure was then piloted with two further PWE. In study documentation and interview guides, we used the term “Accident and Emergency”, as it is often the term used to describe EDs in the UK. Similarly, the term “paramedic” is often used interchangeably to describe ambulance staff, including advanced paramedics and first responders. Where possible, we used participants’ own description of ambulance staff.

### 2.4 Interviews

Twenty-five one-off interviews took place in South East England, between April and September 2019. Interviews were conducted by the lead author (AM), who is a female, postdoctoral researcher with a mixed methods psychology PhD. She has experience of conducting interviews with PWE. Interview times ranged from 28 to 168 minutes (mean: 69). Participants chose their interview location: home, public place, or a university office. Interviews were audio-recorded and transcribed verbatim by a transcription service. Transcripts were anonymised to protect participant confidentiality; they were not returned to participants for comment or correction. Where necessary, quotes with potentially identifying information were edited to preserve anonymity and ensure clarity of meaning. All participants provided written informed consent before their interview. No field notes were taken during interviews. No prior relationships existed between the research team and participants. Participants were aware that the researcher was experienced and interested in epilepsy research, and not affiliated with their usual care provider.

### 2.5 Data analysis

A qualitative framework approach [35] was employed and is suited to policy-oriented research examining patient experiences [36]. Transcripts were read with themes and codes identified. Data were then managed using NVivo (version 12). Following preliminary analysis, two matrices were developed by AM and MM to summarise individual cases, with 7 coded categories relating to: decision-making (self-care, decisions by self/ambulance/public, informing others), care preferences (experiences of using emergency services, satisfaction, suggestions), and 3 categories regarding views and experiences relating to potential ACPs (UTC, ENS, other preferences). Initially, both authors summarised cases to examine consensus, with AM completing coding. A constant comparative approach was used to interrogate the data and identify factors influencing individual participant decisions and preferences. Once complete, a summary of initial findings was presented in October 2019 to a multidisciplinary group of clinicians to elicit their feedback [31].

## 3 Results

Twenty-five people (15 female, 10 male) with an epilepsy diagnosis and frequent seizures participated (see Table 1). Diagnosis length ranged from 4 to 50 years (mean: 21 years). Participants’ ethnicities were White British (n = 22), White other (n = 2) or Black Caribbean (n = 1). Twenty participants said they had someone who helps with their epilepsy (parent: 11, child: 2, partner: 9). Seventeen participants (68%) took 2 or more AEDs. Fifteen participants said they had comorbid health conditions (physical health: 11, mental health: 7). Three reported dissociative seizures. In the past 12 months, participants had seen their GP (n = 13), neurology consultant (n = 20) or ENS (n = 8) for non-emergency epilepsy care.

**Table 1:** Characteristics of respondents.

Characteristics	Sample *n* = 25
**Age bands**	
18–26	5
27–31	7
32–50	6
51–75	7
**Education**	
Other	3
GCSC/o-level	4
A-level	9
Degree	5
Postgraduate	4
**Number of Healthcare professionals seen in past 12 months for epilepsy**	
1	9
2	12
3	3
4	1
**Living situation**	
Alone	5
With others	20
**Tonic clonic seizure frequency (per year)**	
Unclear	5
0	2
1–2	5
3–6	7
12+	6
**Self-report ambulance use (past 12 months)**^a^	
0	1
1–2	15
3–4	5
5–7	3
13	1
**Self-report ED attendance (past 12 months)**^a^	
0	4
1–2	13
3–4	5
5–7	2
12	1
**Strategy used to communicate care needs and/or preferences**	
Medic Alert style bracelet	11
Wallet ID card	7
Mobile App	5
LinkLine device	2
Wearable technology	1

a Note: Eligibility criteria regarding service use was that PWE in the study had visited ED and/or had contact with the ambulance service in the past 12 months for epilepsy.

### 3.1 Decision-making and care preferences after a seizure

Deciding to use emergency services after a seizure was most often due to injury (n = 18) or unusual seizure presentation (n = 15). A small number of participants wished to receive seizure aftercare for reasons which might be judged as not medically necessary. For example, one participant explained that her family contacted the ambulance because they believed she needed oxygen after a seizure. “*I need kind of prolonged oxygen, and not every ambulance carries that, or has a policy of providing it after a seizure… that helps me recover and not go into kind of seizure after seizure*.” NHS ambulance trusts use several vehicle types beyond the traditional double-crewed ‘box’ and ‘van’ ambulance and variation exists in the equipment they carry. The participant said that when a vehicle was sent that did not carry oxygen; they would request an ambulance that did. She felt that if all ambulances stocked oxygen, “*it might reduce the amount of ambulance call-outs*” (p25, F, 40 s).

One participant and his carer explained that being in their 70 s and 80 s meant seizure aftercare was more complex, “*[He] is not very old but he’s getting on and you just sort of hope that the seizures, that’s it, you know, you do want to make sure that he’s in the right place in case it’s anything else.” (p30, F, carer)*.

#### 3.1.1 Seizure characteristics

Losing consciousness may result in a carer calling emergency services for some participants, especially if the seizure type was uncommon. For example, *“If I’m having a fit, a major fit, [my husband] always calls… Because he wants to be on the safe side. And I don’t have these sort of fit very often. I have maybe one or two a year of those big ones” (p3, F, 50 s).* Assessments by ambulance staff sometimes gave reassurance of safety, and participants were therefore reassured that further care was not needed. Where participants remained conscious, many did not wish to receive further care. One participant described an event where an ambulance was called by an emergency service call operator, when he became less responsive during the phone call, *“They just buzzed the ambulance. I cannot really remember” (p27, M, 30 s).* He said on that occasion, ambulance staff arrived to do basic checks, and offered to take him to a hospital, which he declined as he felt it unnecessary after focal seizures.

#### 3.1.2 Seizure location

Seven participants said their carer could intervene and provide support after a seizure at home: “*Most of my seizures are nocturnal, so when [my husband] is near … he knows to put me on my side… he just waits for the seizure to pass” (p2, F, 40 s)*. Most described their carers as being ‘experts on their epilepsy’, sometimes with years of experience, first aid knowledge, or prior guidance from ambulance staff. Carer presence often meant a safety assessment and use of emergency medication. As a result, some calls to emergency services were not thought necessary: “*Mum deals with it as much as she can before having to call paramedics” (p18, F, ≤20)*. However, not all carers were confident. One participant wanted more training options for her husband to provide emergency care, “*[My husband] should be trained more on what to do… they did give me midazolam before that he could put inside of my mouth. But… he’s not going to want to give it to me.” (p29, F, 60 s)*.

Participants described technology that enabled them to receive assistance at home or in public. This included a wearable device for detecting seizures that one participant used to alert a family member: *“If I have a seizure and I am at home alone, because I’ve fortunately, got my device on me that monitors me when I have a seizure, my dad will actually get texts” (p1, M, 20 s).* Several participants said they wanted to utilise technology more to manage their epilepsy. Participants aged over 40 (particularly “young-old”, aged 60–74) were less likely to use these strategies to support their decision-making, *“I didn’t know there were any… it’s never been suggested.” (p30, M, 70 s).* As one participant explained, mobile phone-based apps were wanted but not available, *“They should have a free [app] for people with epilepsy, so that there’s something that I can just press for my husband or a special beep or something to alert people that I’ve had… an attack” (p29, F, 60 s).*

Those without any carer support followed a different process for emergency assistance at home. Two participants used a telephone-based safety alert device called LinkLine. This provided reassurance, as the operator could dispatch an ambulance to their address if there was no verbal response once the alert had been activated.

In public areas, formal protocols of good practice (often held by public transport providers or supermarkets) occasionally influenced decisions about calling emergency services. At times, this conflicted with carer and PWE preference: *“It’s just because he’d had it on the bus so automatically, they had to [call an ambulance].”* This carer felt medical attention was not required: *“[Ambulance staff] just come, check him all over and asked, “Did we want to take him to the hospital?” We said “No.” He’s got no cuts or nothing.” (p7, carer).*

Another participant described being taken to ED despite having uncomplicated seizures at school. She then had a care plan put in place, supported by her usual care providers, that included guidance on appropriate management after a tonic clonic seizure. This plan reduced her subsequent ED use during school hours. Five PWE also said they had a formal care plan with guidance on post-seizure care that was sometimes helpful in managing decisions regarding the need for ED when in public. One participant advocated that care plans are helpful for ambulance staff to be better equipped to provide individualised care: *“If [ambulance staff] were made aware of the relevant details for each person and they were aware of the recovery time and so therefore what to expect of that person, it might be easier for them to communicate, as they’d have more understanding.” (p20, F, 20 s).*

### 3.2 ED alternative care options

When discussing ACPs, several participants said the appropriateness of care options varied on an event-by-event basis. “*There shouldn’t be a set rule of that, yeah, you must go to A&E or yes, you must go to the Urgent Care Centre, it all depends on after doing the observations they do in the ambulance, where they think you should go and also how, depending on how the individual is feeling” (p19, M, 30 s)*.

#### 3.2.1 Experiences of current alternative ED options

When asked if an alternative had ever been offered by ambulance staff, 6 PWE with carer support had previously been offered to remain or be taken home, an option that was described as “safe” and reassuring. A few reported occasions where they continued their journey after ambulance staff assessment, as one participant recalled: *“I remember in the past being, you know, saying that ‘You must go’ and I’m saying, ‘No, I can’t, I don’t need to.’”* She was therefore thankful to carry on with her day after she had recovered from a seizure and described having *“… a very good experience because somehow they realised what I wanted, and took me to [location redacted] which is where I was going, which was incredible, because that meant me not having to be hours in A&E, and the worry of somebody coming to meet me and things like that” (p5, F, 50 s).*

One participant with frequent seizures in public, had regular contact with the local ambulance service, who were aware of his preference to avoid ED: *“As soon as you put my name into the system, it flags. They know it’s related to me or even certain descriptions... They know ‘Okay, this is what we do… if we can try and keep you in the community, that’s what we’ll do. We’ll try and keep you away from hospital as much as possible’” (p1, M, 20 s).*

Some participants said other care attributes like medication advice and ED follow-up were more important than an ACP. One participant had been diagnosed with epilepsy 6 years prior and had 5 tonic clonic seizures in a short space of time: *“I’d like to get scheduled back in with a neurologist because the last time I saw a neurologist was… the initial diagnosis.” (p12, M, 20 s).* Many preferred to see their GP for a general follow-up after ED, *“An appointment should be booked with the GP afterwards as like a follow-up service… What it should be is it should be classed as an emergency appointment in a few days or … they should call it like an A&E follow-up” (p20, F, 20 s).* Some said they wanted a follow-up by someone with epilepsy-specific knowledge and others wanted a mechanism in place whereby an ED visit would trigger some form of letter or appointment booking from their usual care providers to check-in with the patient.

#### 3.2.2 Views about an Urgent Treatment Centre as an alternative to ED

Four participants preferred to avoid ED unless they felt there was no other option and were therefore supportive of attending UTCs instead. Some were favourable of UTCs due to perceived ease of access, as one participant explained, *“That would be nice to know if there was plenty of the walk-in centres around, that if I did have a seizure outside in public or something, even with my family, if I do cut myself or put myself in danger in some way or another, that if it’s really close by, I could go to that” (p7, M, 20 s).*

Overall, UTCs were however not seen as a replacement for ED, mainly due to limited opening hours. Several participants had nocturnal seizures, *“They’re only open* 12 h *a day. So, if I had the seizure at night, it wouldn’t be helpful” (p25, F, 40 s).* Others were concerned that their visit might run overtime and ED use would occur anyway: *“If I had a seizure at… [Laughter] well, like, 4:30pm and they closed at 6:00pm, would that be the best idea?” (p17, M, 20 s)*. Three participants expressed worry about transportation after a seizure: *“My only query would be would an ambulance take me there? Would I have to get there?” (p26, F, 30 s).*

#### 3.2.3 Views about an Epilepsy Nurse Specialist phone call as an alternative to ED

Coupled with mortality fears, participants who lived alone felt positively about having a scheduled phone call for support and monitoring, *“Sometimes, to be honest, I was lying in the bed and I don’t know if now I’m going to wake up or not, you know. But if somebody called then, definitely, going to wake up.” (p6, F, 20 s).* ENSs were viewed as having specialist knowledge that provided reassurance: *“The epilepsy nurse specialist, have been a great help to me in the past… to have their reassurance and their knowledge, expertise… that would be most, you know, comforting, really” (p10, F, 50 s).* ENS support could be beneficial if they had prior rapport and access to individual patient history: *“I’d want it to be my epilepsy nurse because otherwise… they’re not going to be able to know my epilepsy in the same way.” (p20, F, 20 s).*

Despite positive appraisal of ENS access, most participants were concerned that this option was not a replacement for ED, but an adjunct to usual care. Some described speech difficulties after a seizure that would make communication over the phone difficult. The practicality of a phone call was raised by participants who lived alone, *“If someone was at home alone… and they were having more seizures, I mean, would they be contactable by phone?” (p10, F, 50 s).* Others described concerns with ENS lack of availability and capacity, based on their previous experience of engaging with ENSs for their usual care: *“There’s a sort of 15-minute wait until you get through to them” (p25, F, 40 s).* One participant suggested systemic issues might prevent ENSs from delivering their service as an ACP: *“There’s not enough of these epilepsy nurses, they’re very, very busy, because more people have got the epilepsy now.” (p29, F, 60 s).*

## 4 Discussion

We recruited a group of highly informed PWE and their carers. Their decision-making was mostly concordant with medical guidelines, where injury or unusual seizure presentation are indicators for ED attendance [11]. Participants reported high seizure frequency and AED use compared with national averages [15,17], characteristic of uncontrolled epilepsy.

We found evidence of unwanted and/or unnecessary use of emergency services following a seizure. Erroneous beliefs on the need for ED and treatments raised by several participants, such as routine access to oxygen following a seizure, might be addressed through improving knowledge and education. More than half of participants experienced frequent seizures in public, which may partially explain some instances of unwanted emergency service use. Decision-making in public has been well-described elsewhere [37], but we found participants tried to triage themselves away from ED, retaining autonomy by using preventative measures for seizure-management when in public or alone. Many prepared these strategies to augment decision-making through use of ID cards and medical alert bracelets. Care plans may also enhance patient autonomy by issuing guidance to others on seizure aftercare [38,39]. We found variable use of care plans amongst the group, despite UK guidelines [40], and being routine in paediatric epilepsy [41] and other LTCs like diabetes [42].

The prehospital assessment that occurs on scene is critical for giving patients reassurance [43] and facilitating sustainable, effective use of ED [44]. UK ambulances operate with systemic restrictions [23] and limited guidance on seizure management [25], which means ED conveyance can be at times, unavoidable [23]. Implementing resources to support ambulance staff decision-making was highlighted by participants in this study. Their proposals included increasing access to basic health status (i.e., diagnosed conditions, medications, allergies) and patient care plans.

It is important to note that since our interviews took place, changes have been made to guidance for ambulance staff in the UK on managing seizures (via the Joint Royal Colleges Ambulance Liaison Committee [45]). There has been some increase within this document to the issue of when it is and is not appropriate to consider non-conveyance for those presenting with seizures. Ambulance staff have previously identified limited guidance as a barrier to non-conveyance [25]. It remains to be determined to what extent the guidance update will change practice [46]. Dispatch centres could further support ambulance staff [25,47] by helping to differentiate a first seizure from established epilepsy [48]. Several participants were known to local emergency services and their care was based on prior history; however, this was not generally the case.

Some participants tried to improve communication of their individual care preferences through digital technology. However, while these might be better utilised in the future to improve quality of patient care and reduce service costs [49], a number of barriers to the widespread uptake of digital solutions have been identified. This includes lack of efficacy data, patient non-adherence and confidentiality concerns [50]. One specific concern regarding the use of wearable technology is the risk of false positives in seizure detection, which can reduce reported benefits [51]. There is also a lack of awareness of effective technology [52], as highlighted by several participants in our study.

We presented participants with two hypothetical ACP options during interviews: a visit to Urgent Care instead of ED, or remote, telephone-based support from a specialist nurse. To our knowledge, only one group have published an epilepsy ACP pilot to date [53]. Authors evaluated ENS follow-up 5 days after a seizure, and although positive outcomes were reported, uptake for this pathway was low. Our findings highlight the value many patients receive from ENS support [25,54] but concerns were raised in conducting a safety assessment over the phone in an emergency when patient background and individual needs are not known. Other major concerns were identified around UTC ability to accept patients outside of working hours or to manage a deterioration in status.

Participants preferred ENS support as part of their usual care, and proactive follow-up after ED attendance. Such follow-ups with a GP or health professional with epilepsy knowledge rarely occurred after ED, despite being a potentially cost-saving and preventative approach [12]. Participants wanted a mechanism, whereby, all patients are linked back in with preventative services, that could provide specialist advice and training to support self-management, potentially reducing unnecessary or unwanted ED use.

### 4.1 Strengths and limitations

Participants were drawn from Southeast England and many recruited through a user-group charity. They were likely to be more educated about epilepsy, characterising early adopters of innovation [55]. It should be noted that recruitment methods may have led to an underrepresentation of PWE who prefer contact with emergency services after an uncomplicated seizure and use ED on a regular, monthly basis. We recruited participants who self-reported contact with emergency services for epilepsy in ≤12 months., Their epilepsy was more severe than the national average, as over half of PWE in the UK have no seizure activity in the past year [17]. They were a group who had innovative, well-developed strategies for managing seizures at home and alerting services that might be implemented more widely. Interviews were detailed, yielding insightful qualitative data, particularly about those who triaged themselves away from emergency services to prevent unwanted ED attendance.

### 4.2 Conclusions

Our findings reinforce the complexity of emergency seizure care and the importance of proactive, collaborative partnerships between patients and service providers to improve outcomes. Themes from this study emphasised the importance of information sharing between services to provide patients with high-quality, individualised care. Although most participants did not favour either hypothetical ACP presented, some described scenarios where options could be beneficial, particularly when specialist knowledge and real-time monitoring were accessible.

## Funding

This project is funded by the National Institute for Health Research’s Health Services and Delivery Research Programme (project number 17/05/62). The views and opinions expressed herein are those of the authors and do not necessarily reflect those of King’s College London, the University of Liverpool, the HS&DR programme, the NIHR, the NHS, or the Department of Health and Social Care.

## Ethical approval

Ethical approval provided by the King’s College London’s Psychiatry, Nursing and Midwifery Ethics Committee (LRS-18/19-10353).

## Declaration of Competing Interest

The authors declare that they have no known competing financial interests or personal relationships that could have appeared to influence the work reported in this paper.
