# Supplementary material for: Patient views on use of emergency and alternative care services for adult epilepsy: A qualitative study
Source: Seizure. 2020 Aug;80:56–62. doi: 10.1016/j.seizure.2020.04.011 (PMC7443693; doi:10.1016/j.seizure.2020.04.011)
Supplement: Supplementary file 1 [file mmc1.docx]

# Supporting Information A Definitions

1. Epilepsy Nurse Specialist Definition

*An epilepsy nurse is a registered nurse who typically has extra experience or qualifications in neurology.* *Their role varies but they can provide information regarding epilepsy and its management. They can help monitor and change medication, order tests and act as a first point of contact for patients and general practitioners (primary care doctors). They advise people in a variety of settings, including in outpatient clinics and over the phone.*

1. Epilepsy Nurse – Proposed Alternative Care Pathway*

*“Another option that is being considered instead of always taking people with epilepsy to A&E following a seizure, is for the person to be left at home or taken home if they were out. They would then be telephoned within say 24 hours by an epilepsy nurse specialist. The nurse would be phoning to see how the person was recovering and whether needed any additional support, such as a change in medication, advice or to be booked in to see a neurologist. “*

1. Urgent Treatment Centre Definition

*Urgent Treatment Centres are operated by general practitioners (primary care doctors) in the community. Usually, they will accept walk-in bookings by patients with minor health concerns where use of the Emergency Department is not required.*

1. Urgent Treatment Centre – Proposed Alternative Care Pathway*

*“The term Urgent Treatment Centres is a relatively new one, but it has been suggested as a potential alternative to A&E. To give you a feel of what they are, the idea is that: -They will be open at least 12 hours a day, 7 days a week. Staffed by GPs and nurses. -Be able to issue prescriptions and have access to some common, but basic equipment, such as ECGs to test heart function and in some cases X-ray machines. How many there are new you and where they are varies depending on where in the country you are. Some are located next to GP practices, some are on hospital sites. - You might be more familiar with terms like ‘walk-in centres’ and ‘minor injury units’. These are to be relabelled urgent treatment centres.”*

*Described during participant interviews
