# Supplementary material for: Patient views on use of emergency and alternative care services for adult epilepsy: A qualitative study
Source: Seizure. 2020 Aug;80:56–62. doi: 10.1016/j.seizure.2020.04.011 (PMC7443693; doi:10.1016/j.seizure.2020.04.011)
Supplement: Supplementary file 2 [file mmc2.docx]

# Supporting Information B – Inclusion and Exclusion Criteria

Box 1. Eligibility Criteria

| Participant | | Carer | |
| --- | --- | --- | --- |
| Inclusion: | Exclusion: | Inclusion: | Exclusion: |
| - Established diagnosis of epilepsy (1+ year) - All epilepsy syndromes and all types of focal and generalised seizures - Currently being prescribed antiepileptic medication - Aged 18 years or older (no upper age limit) - Have visited ED and/or had contact with the ambulance service in the past 12 months for epilepsy (self-report) - Live in the London/ South-east area of England - Able to provide informed consent and participate in a qualitative interview in English | - Severe current psychiatric disorders (e.g. acute psychosis) or life-threatening medical illness - Resides within a care or nursing home or has no fixed abode. | - A significant other to the patient (e.g., family member, friend) who the patient identifies as providing informal support - Aged 18 years or older (no upper age limit) - Lives in London/ South-east area of England - Able to provide informed consent and participate in a qualitative interview in English | - Severe current psychiatric disorders (e.g. acute psychosis) or life-threatening medical illness - Resides within a care or nursing home or has no fixed abode. |
|  |  |  |  |
|  |  |  |  |
|  |  |  |  |
|  |  |  |  |
|  |  |  |  |
