# Supplementary material for: Patient views on use of emergency and alternative care services for adult epilepsy: A qualitative study
Source: Seizure. 2020 Aug;80:56–62. doi: 10.1016/j.seizure.2020.04.011 (PMC7443693; doi:10.1016/j.seizure.2020.04.011)
Supplement: Supplementary file 3 [file mmc3.docx]

# Supporting Information C – Interview Guide

Box 2. Interview Topic Guide

1. Could you share with me your experiences with emergency services that may have led you to being interested in this study?
2. Do you have a someone who helps with your epilepsy? If yes, How often have they been with you when you’ve had seizure in last 12 months? How confident do they feel in knowing what to do when a seizure happens?
3. What is your impression of how many seizures you have? (Prompt: i.e., in the last year, month, year?)
4. How would you describe your seizures? (Prompt: what kind do you usually have?), How long have you been diagnosed with epilepsy?
5. Medication for seizures: What do you take for your seizures? How do you feel about managing your seizures?
6. Are there any triggers or warnings before the onset of your seizures?
7. How many times have emergency services been called in the past 12 months? How many of those times have you gone to A&E?
8. how often would you have seizures in public versus at home? Can you think of a recent example and tell me where this was? Were you by yourself or with others (who?) *What type of seizure did you have on that occasion? Were you unconscious at any point?* Was an ambulance called *(If so, by whom?)*? What did *the paramedics* do? (i.e., *assess,* take to A&E) Did you have any chance to discuss where you would like to go?
9. Have you ever not been taken to A&E after seizure in a public place? (Prompt: tell me more about what happened then. i.e., who was present, was this what you wanted?) *Have you ever found that paramedics were aware of your care plan and/or medication history at the time of the event? If yes, can you tell me more about that?*
10. Do you have any emergency devices, or a medic alert jewellery?
11. What do you do if a seizure happens when you are alone and no one else is around? Have you called an ambulance for yourself when you’ve had a seizure?
12. When someone else has called an ambulance after you’ve had a seizure, who typically makes the decision to call? Who would typically make the decision for you to go to A&E?
13. Can you tell me more about what your general experiences of Emergency Services for your epilepsy?
14. When was the last time you used emergency services and didn’t use A&E? What happened?
15. What kinds of things *influence whether or not you would like to receive care by* emergency services? (i.e., *injury,* location, seizure type)
16. Can you tell me about the last time you went to A&E for your epilepsy? Would you say this is typical for you? Prompts if needed: Why did you go to A&E? Who made decision/organised E.g., carer/relative, other lay people, ambulance staff, police, etc.
17. If you had to use emergency services again, are there any changes that you would like to see in the way this service is provided? (Prompt if needed: and how about A&E?)
18. What do you believe is most helpful about going to A&E? E.g., Reassurance, confidence, treatments available, reduce burden on others. (write down/ *probe)*
19. *If/when patients mention reassurance, ask about factors that they perceive to provide this, so we can see how these overlap with other, more concrete attributes (e.g., type of care provider, tests available etc.)*
20. *Which aspects of the care you receive are the most important to you?*
21. Do you see there being anything negative about going to A&E? E.g., Time, not necessary, unnecessary tests and examinations, disrupted rest, don’t like hospitals/being patient, etc. (write down/ *probe)*
22. What has been your experience of the link-up/ communication between A&E and your usual care provider? *(probe for clarification)*
23. Have your usual care providers been informed of A&E visits?
24. Have you been contacted and offered any extra support because you had been to A&E?
25. When you have had a seizure and contact has been made with emergency services (i.e., 999/paramedics/111), have you ever been offered an alternative to A&E? If so, what?
26. If you weren’t taken to A&E, where would you like to be taken once your seizure had finished? What health professional would you like to see at the time? *Are there any specific tests and equipment you would want to see or is it just the possibility of being able to access what is needed, just in case?* What kind of follow-up would you like to have?
27. Are you familiar with something called an Urgent Treatment Centre? (explanation). What might be your expectations of such a centre be? Would you have any concerns about this? If so, what kind? Would anything change how you felt about this ACP? I.e.., seizure type, recent changes in meds.
28. Another option that is being considered instead of always taking people with epilepsy to A&E following a seizure, is for the person to be left at home or taken home if they were out. They would then be telephoned within say 24 hours by an epilepsy nurse specialist. The nurse would be phoning to see how the person was recovering and whether needed any additional support, such as a change in medication, advice or to be booked in to see a neurologist. What might be your expectations be over this sort of alternative to being taken to A&E? Would you have any concerns about this? If so, what kind? Would anything change how you felt about this ACP? I.e.., seizure type, recent changes in meds

Note. A&E: Accident and Emergency care (term used to refer to UK-based Emergency Department). Questions and prompts in italics were added in July 2019 to reflect additional probes on interview topics of interest.
